# Supplementary material for: IL1R1+ cancer-associated fibroblasts drive tumor development and immunosuppression in colorectal cancer
Source: Nat Commun. 2023 Jul 17;14:4251. doi: 10.1038/s41467-023-39953-w (PMC10352362; doi:10.1038/s41467-023-39953-w)
Supplement: Supplementary file 1 — Supplementary Information [file 41467_2023_39953_MOESM1_ESM.pdf]

SUPPLEMENTARY INFORMATION Koncina et al.

IL1R1+ cancer-associated fibroblasts drive tumor development and immunosuppression in colorectal cancer

Koncina E.[1*], Nurmik M.[1*], Pozdeev V.I.[1*], Gilson C.[1], Tsenkova M.[1], Begaj R.[1], Stang S[2]., Gaigneaux A.[1], Weindorfer C.[2], Rodriguez F.[1], Schmoetten M.[1], Klein E.[1], Karta J.[1], Atanasova V.S.[2], Grzyb K.[3], Ullmann P[1]., Halder R.[3], Hengstschläger M.[2], Graas J.[4], Augendre V.[5], Karapetyan Y.[6], Kerger L.[7], Zuegel N.[7], Skupin A.[3], Haan S.[1], Meiser J.[8], Dolznig H.[2$], Letellier E.[1$#]

[1]Molecular Disease Mechanisms Group, Department of Life Sciences and Medicine, University of Luxembourg, Belval, Luxembourg
[2]Center for Pathobiochemistry and Genetics, Medical University of Vienna, Austria
[3]Luxembourg Centre for Systems Biomedicine, University of Luxembourg, Belval, Luxembourg
[4]Clinical and Epidemiological Investigation Center, Department of Population Health, Luxembourg Institute of Health, Luxembourg
[5]National Center of Pathology, Laboratoire National de Santé, Luxembourg
[6]Integrated BioBank of Luxembourg, Dudelange, Luxembourg
[7]Department of Surgery, Centre Hospitalier Emile Mayrisch, Esch-sur-Alzette, Luxembourg
[8]Cancer Metabolism Group, Department of Cancer Research, Luxembourg Institute of Health, Luxembourg, Luxembourg

$correspondence to: Elisabeth Letellier, elisabeth.letellier@uni.lu and Helmut Dolznig, helmut.dolznig@meduniwien.ac.at

#lead author: Elisabeth Letellier, elisabeth.letellier@uni.lu

***These authors contributed equally**

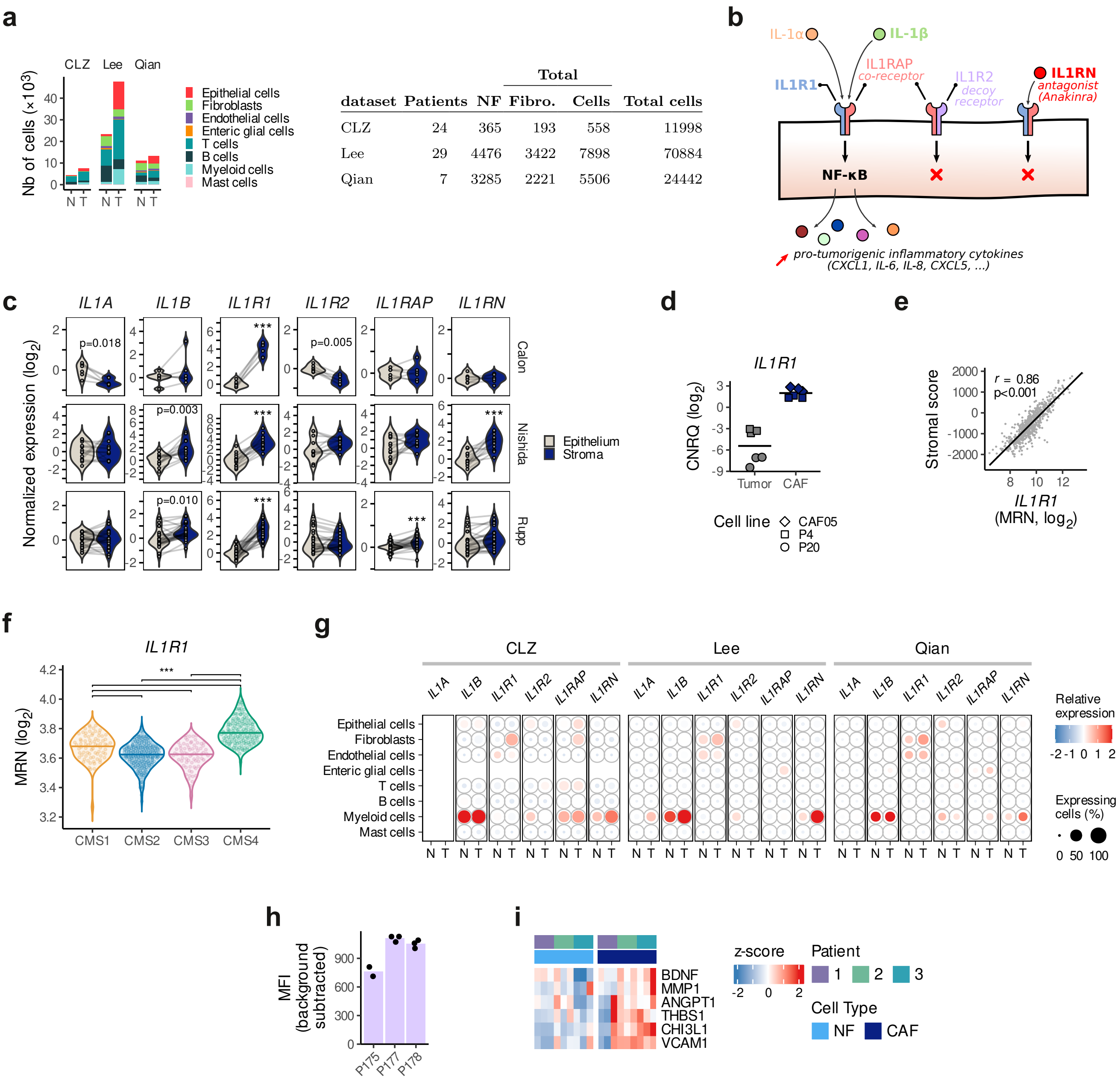

Supplementary Figure 1 – Expression of the IL1 pathway members in CRC. a Proportion of cells in normal and tumor tissue in each dataset (left barchart) and number of total cells as well as normal and tumor fibroblasts identified in each of the 3 scRNA-Seq datasets (right table). **b** Components of the IL-1-family showing the three receptors IL1R1, IL1RAP and IL1R2 including their functional characteristics, both ligands IL-1α and IL-1β as well as the IL1RN antagonist. **c** Gene expression of IL-1-family members in tumor stroma and tumor epithelium in the Calon (n=6), Nishida (n=13), and Rupp (n=26) datasets. Statistical differences were determined using two-sided paired t-tests with Holm's correction for multiple comparisons (***, p < 0.001). **d** *IL1R1* expression in primary cultures of patient derived CAFs and tumor spheroids (from P4 and P20, as well as the commercially available CAF05 cell line). Values represent calibrated normalized relative quantities (CNRQ, $\log_2$). **e** Correlation between the ESTIMATE stromal score and *IL1R1* expression in the n=624 human TCGA CRC samples. The Pearson's coefficient r and p-value are shown. **f** Expression of *IL1R1* in TCGA CRC patients (n=510) according to their CMS subtype. Statistical differences were determined using an ANOVA followed by Holm's adjusted two-sided pairwise t-tests (***, p < 0.001; brackets show the significantly different pairs). The horizontal lines show the median. **g** Expression of IL-1 family genes in the different cell types from normal (N) and tumor (T) tissue in the three scRNA-seq datasets (CLZ, Lee and Qian). The heatmap color gradient shows the relative expression (mean of scaled normalized counts) and the bubble size shows the percentage of expressing cells. **h** IL1R1 expression measured by flow cytometry in CAF cultures isolated from 3 distinct patients (characteristics in Supplementary Table 3). **i** NFκB target genes expression in NFs and CAFs from the GSE198697 dataset (matched NF/CAF cultures from n=3 patients). Number of patients per dataset in panels a and g is reported in Supplementary Figure 1a. Source data are provided as a Source Data file.

Supplementary Figure 2

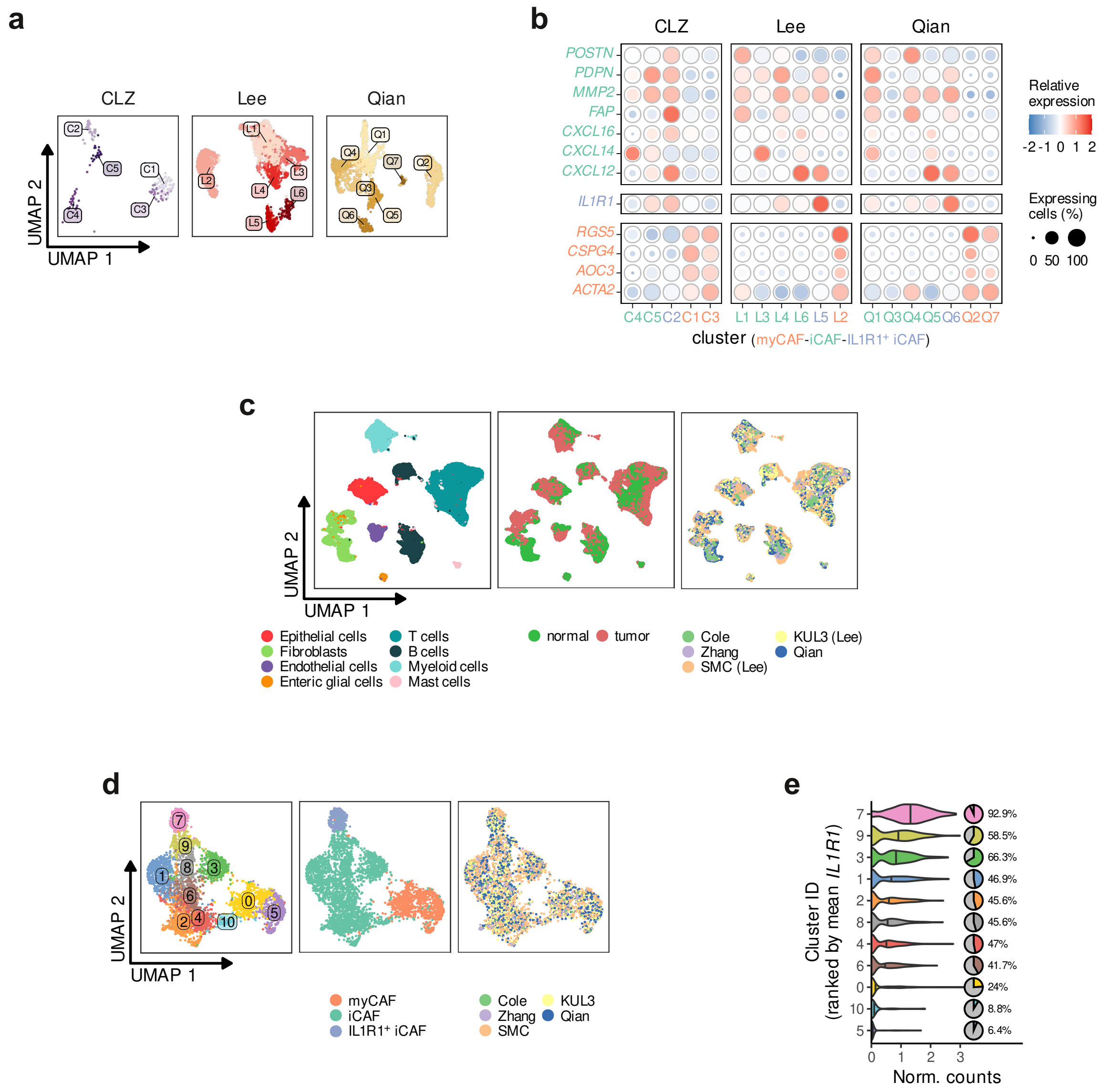

Supplementary Figure 2 – Single cell sequencing analysis of CAF subtypes in CRC. a UMAP plot showing the individual cell clusters identified in CAFs from the three scRNA-seq datasets (CLZ, Lee and Qian). **b** The different clusters were grouped into myCAF and iCAF based on the expression profile of canonical markers. Expression of *IL1R1* was used to further distinguish IL1R1⁺ iCAFs from the remaining iCAF clusters. The heatmap color gradient shows the relative expression (mean of scaled normalized counts) and the bubble size the percentage of expressing cells. **c** UMAP plot showing the main cell types identified in the integrated (Cole, Zhang, Lee-SMC, Lee-KUL3 and Qian) dataset (left) and the distribution of normal and tumor tissue (middle) as well as the distribution of the cohorts composing the dataset (right). **d** UMAP plot showing the identified CAF clusters (labelled 0-10) in the integrated dataset (left), the identified iCAF, IL1R1⁺ iCAF and myCAF group of clusters (middle) and the distribution of the cohorts composing each cluster (right). **e** Violin plot showing *IL1R1* expression and pie charts showing the percentage of *IL1R1* expressing cells in each CAF cluster. Colors represent different clusters and the same color code was used in **d** and **e**. Number of patients per dataset in panels a,b is reported in Supplementary Figure 1a. Source data are provided as a Source Data file.

Supplementary Figure 3

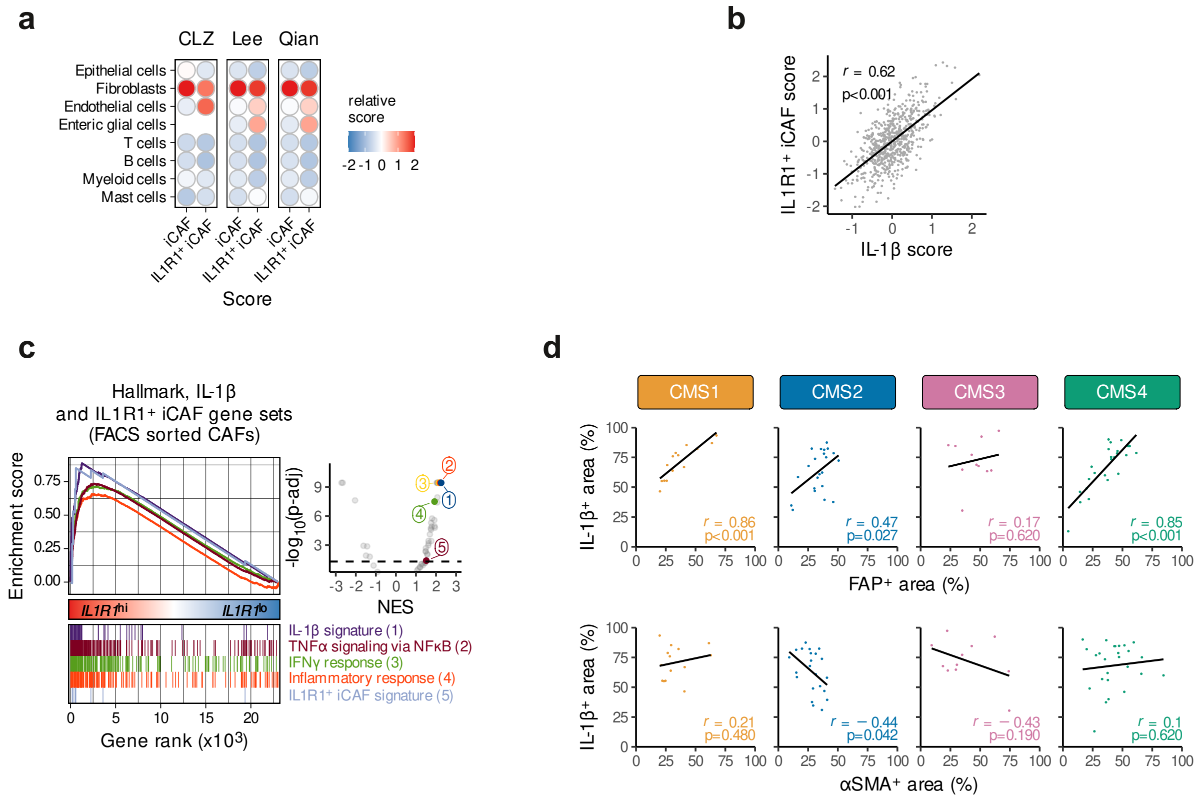

Supplementary Figure 3 – Characterization of the IL1R1⁺ iCAF subtype in CRC. a Heatmap showing iCAF and IL1R1⁺ iCAFs scores (z-score) in the main cell-types of the three scRNA-seq datasets (CLZ, Lee and Qian, Number of patients per dataset is reported in Supplementary Figure 1a). **b** Correlation between the IL-1β and IL1R1⁺ iCAFs scores in the n=624 human TCGA CRC samples. The Pearson's coefficient r and p-value are shown. **c** GSEA of genes expressed differentially between FACS-sorted IL1R1^{hi} and IL1R1^{lo} CT5.3 cells, n=3 independent experiments. The MSigDB Hallmark gene set extended with our IL-1β and IL1R1⁺ iCAF gene sets were used. Running enrichment scores (ES) of selected gene sets are shown in addition to the volcano plot showing the normalized enrichment scores (NES) and adjusted p-values for all tested gene sets. **d** Correlation between FAP⁺ and IL-1β⁺ staining (upper panel) and αSMA⁺ and IL-1β⁺ staining (lower panel) after IHC staining on tissue microarray sections of our established in-house CRC cohort and split by CMS (n=73 patients with identified CMS out of the total of 106 available TMAs). Source data are provided as a Source Data file.

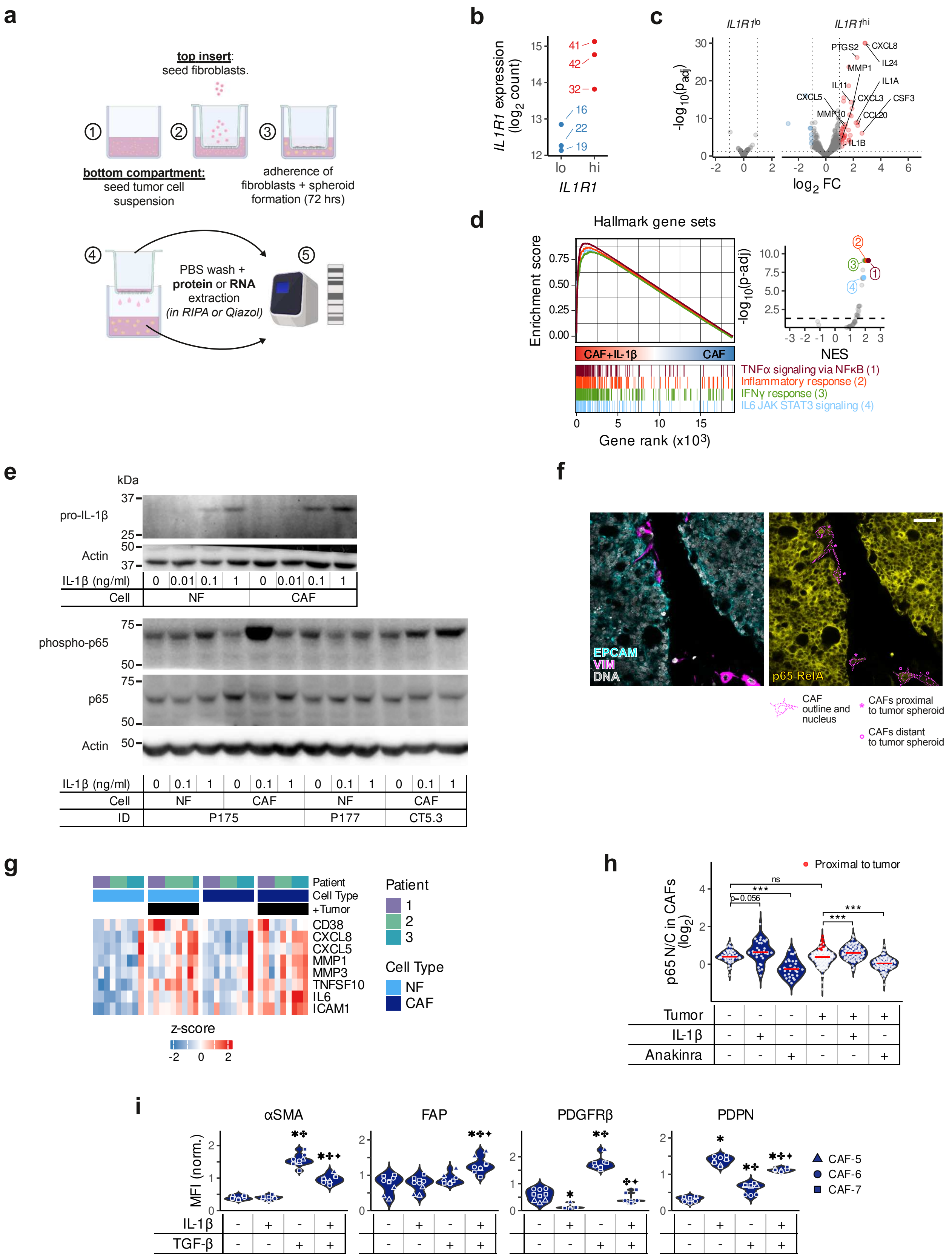

Supplementary Figure 4 – Pro-tumorigenic effect of the IL1R1⁺ iCAF in CRC. a Experimental layout of transwell CAF-tumor co-cultures. Tumor spheroids are seeded in 6-well ultra-low attachment (ULA) plates (1), after which a transwell insert is added and CAFs are seeded into the top compartment (2). After cultivation for 72 hours (3), both compartments can be lysed and collected separately (4) allowing to identify the effect of the co-culture on both cell types independently (5). **b** *IL1R1* expression in CAF cultures from six different patients (in-house cohort) classified into *IL1R1*^{hi} (n=3 independent biological replicates from three different patients P32, P41 and P42) and *IL1R1*^{lo} (n=3 independent biological replicates from three different patients P16, P19 and P22) based on the median expression level, as assessed by RNA-seq. **c** Volcano plot of genes differentially expressed upon co-culture of either *IL1R1*^{lo} (P16, P19, P22) or *IL1R1*^{hi} (P32, P41, P42) CAFs with HT-29 tumor spheroids. **d** GSEA of genes differentially expressed in CAFs upon treatment with IL-1β (1 ng/ml). The running enrichment score (ES) for selected gene sets

is shown in addition to the volcano plot showing the normalized enrichment score (NES) and adjusted p-values of all 50 MSigDB Hallmark gene sets. **e** p65, phosphorylated p65, pro-IL-1β and Actin expression in NFs and CAFs (P4) upon IL-1β stimulation (0.01, 0.1 and 1 ng/ml), as assessed by western blotting in two independent experiments with multiple NF/CAF pairs. **f** EPCAM, Vimentin and p65-RelA staining on LS174T tumor organoids and CAF-8 cells. The outlines of CAFs as performed to measure p65-RelA staining intensity are shown (pink outlines on the right panel). CAFs considered proximal (asterisk) and distal (open circles) to tumor organoids are highlighted. Representative experiment out of two independent experiments (Figure 3g). Scale bar = 50 µm. **g** Heatmap showing NFκB target genes in NFs and CAFs cultured alone or in presence of tumor organoids in GSE198697 (matched NF/CAF cultures from n=3 patients). **h** p65 nuclear-to-cytoplasmic ratio (N/C) in CAFs. After treating tumor cell (LS174T) – CAF (CAF-7) co-cultures with either IL-1β or Anakinra, ICC staining of p65 was quantified using ImageJ and N/C was calculated. Red dots show CAFs in close proximity to tumor spheroids (<25 µm). Tukey post-hoc test following an ANOVA (***, $p < 0.001$; ns, $p \geq 0.05$). Representative experiment out of two independent experiments (Figure 3g) **i** CAF phenotype induced by IL-1β and TGF-β activation crosstalk. CAFs (primary cultures of CAF-5, CAF-6 and CAF-7, patient characteristics in Supplementary Table 3) were treated with either IL-1β (5 ng/ml), TGF-β (5 ng/ml) or both cytokines together and the expression of PDGFRβ, FAP, αSMA and PDPN measured by flow cytometry. MFI values obtained on the three different CAFs were normalized (non-centered scaling). Different data point shapes show technical replicates for the three different CAFs. Tukey post-hoc test following a nested ANOVA design (✱/✤/✦: $p < 0.001$; ✱ vs. untreated control, ✤ vs. IL-1β treated and ✦ vs. TGF-β treated). Source data are provided as a Source Data file.

Supplementary Figure 5

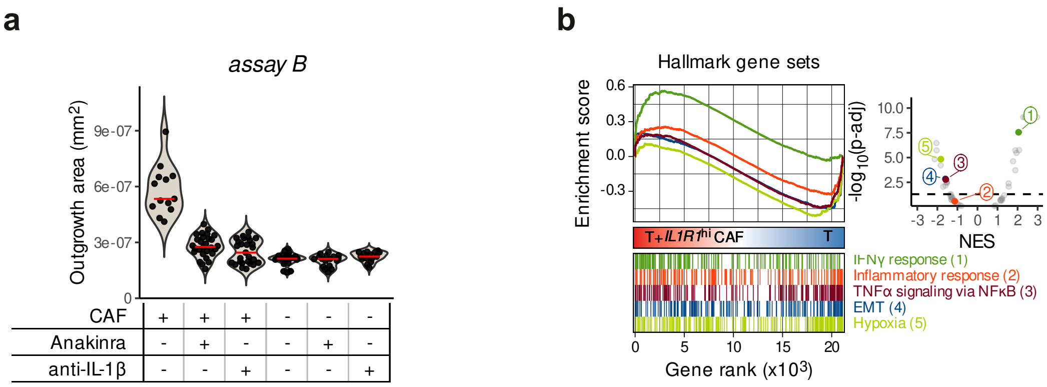

Supplementary Figure 5 – IL1R1^{+} iCAF subtype-induced signaling pathways in tumor cells. a Organotypic encapsulation assay (*assay B* in Fig. 4A) where tumor spheroids (P4) were encapsulated either alone or with CAFs (CT5.3) and treated with anti-IL-1β (100 ng/ml) or Anakinra (100 ng/ml). One representative experiment out of two is shown. **b** MSigDB Hallmark GSEA in tumor spheroids (HT-29) upon co-culture with *IL1R1*^{lo} CAFs. The running enrichment score (ES) for selected gene sets is shown in addition to the volcano plot showing the normalized enrichment score (NES) and adjusted p-values of all 50 MSigDB Hallmark gene sets. Source data are provided as a Source Data file.

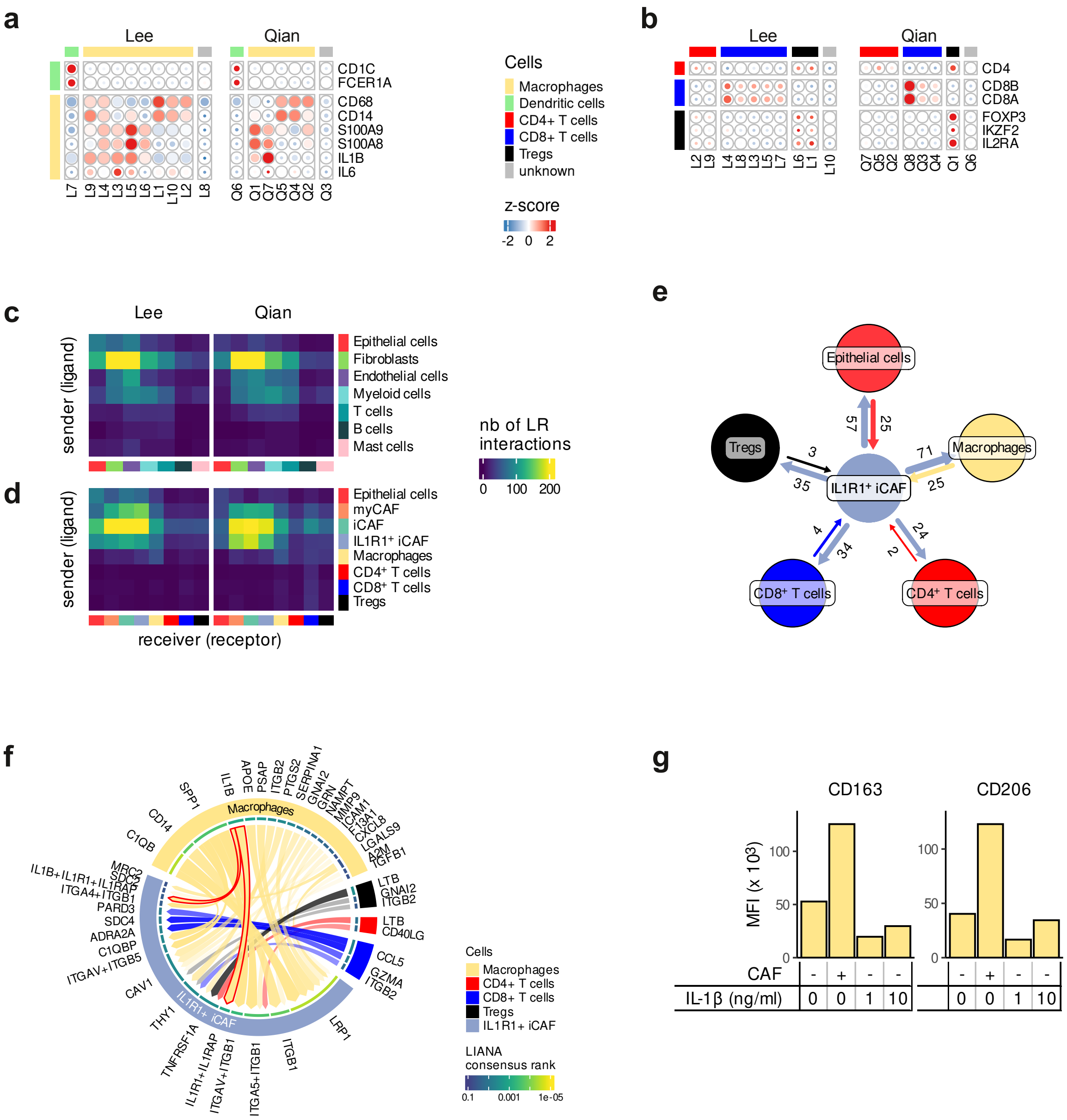

Supplementary Figure 6 – IL1R1⁺ iCAFs immune cell interaction analysis. a,b Tumor myeloid in **a** and T cells in **b** in the Lee and Qian scRNA-Seq datasets were subclustered and labeled based on the expression profile of canonical markers. The heatmap color gradient shows the relative expression (mean of scaled normalized counts) and the bubble size the percentage of expressing cells. **c,d** Ligand-receptor (LR) interactions detected by LIANA between main cell compartments in **c** or between selected subsets in **d** (CAFs, macrophages/monocytes, epithelial cells and T cells) in the Lee and Qian datasets. The heatmap shows the number of distinct LR pairs identified in each cell sender-receiver combination. **e** Potential LR interactions between IL1R1⁺ iCAFs and epithelial cells, macrophages and T cells detected by LIANA in the Qian scRNA-seq dataset. **f** Chord diagram showing LR interactions (LIANA aggregate score < 0.05) between ligands borne by immune cells (macrophages, CD4⁺ T cells, CD8⁺ T cells and Tregs) and receptors borne by IL1R1⁺ iCAFs in the Qian scRNA-seq dataset. Arrow thickness and opacity shows higher ranked LIANA scores. Arrows outlined in red highlight the IL1β-IL1R1 pair. **g** Expression of CD163 and CD206 (MFI) on PBMC derived macrophages treated with either 1 or 10 ng/ml IL-1β, or grown in the presence of CAF-3 cells. One representative experiment out of two is shown. Number of patients per dataset in panels a-f is reported in Supplementary Figure 1a. Source data are provided as a Source Data file.

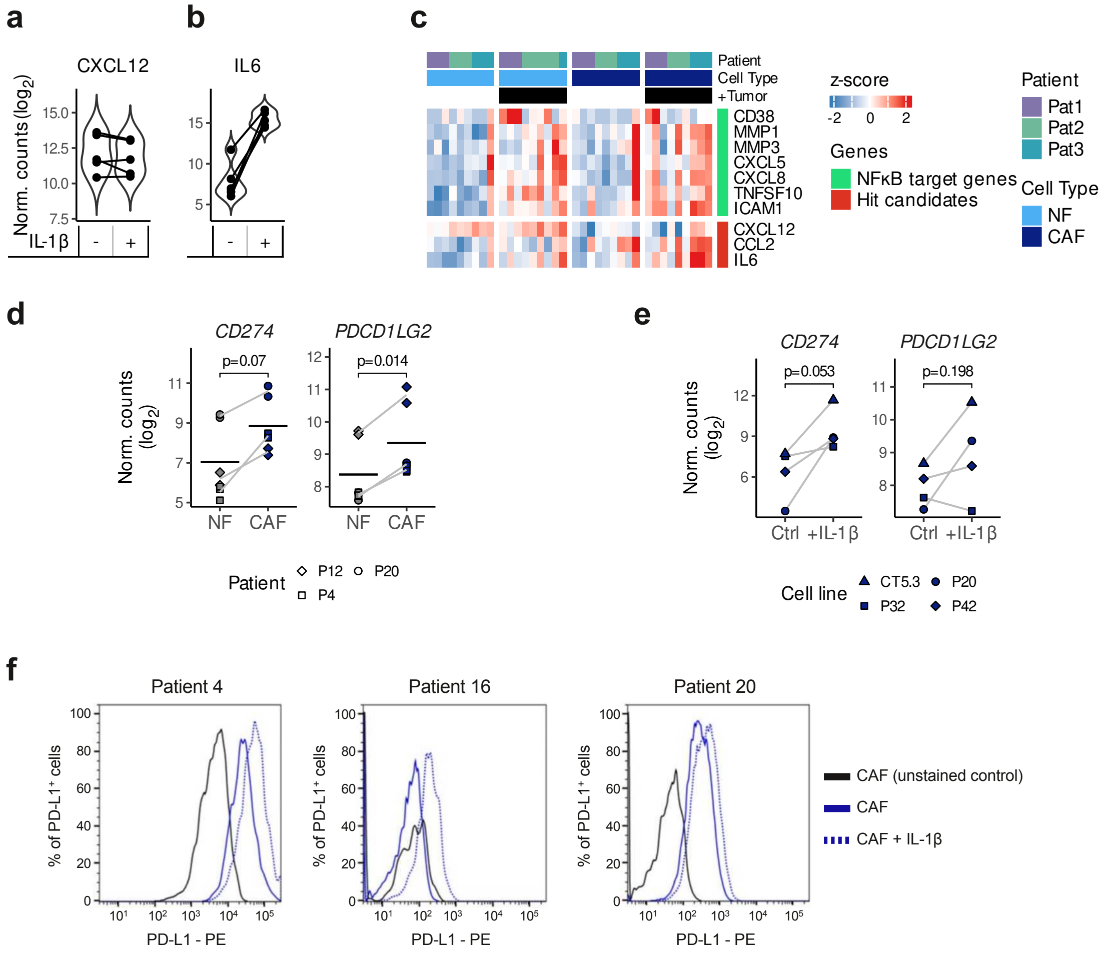

Supplementary Figure 7 – IL-1β induced signaling in CAFs. a,b Violin plot showing the expression of *CXCL12* in **a** and *IL6* in **b** upon stimulation of fibroblasts with IL-1β. **a,b** represent five independent CAF cultures. **c** Heatmap showing the expression of identified hit candidates and NFκB target genes in NFs and CAFs upon coculture with tumor organoids (GSE198697, matched NF/CAF cultures from n=3 patients). **d** Expression of *CD274* (PD-L1) and *PCD1LG2* (PD-L2) in paired NFs and CAFs (P4, P12, P20) in bulk RNA-seq data. P-values from paired t-tests are shown. **e** Expression of *CD274* (PD-L1) and *PCD1LG2* (PD-L2) in CAFs (P20, P32, P42, and CT5.3) after IL-1β stimulation (1 ng/ml) in bulk RNA-Seq data. P-values from two-sided paired t-tests are shown. **f** Presence of surface-bound PD-L1 protein assessed via flow cytometry in control CAFs (P4, P16 and P20) and after stimulation with IL-1β (1 ng/ml). Source data are provided as a Source Data file.

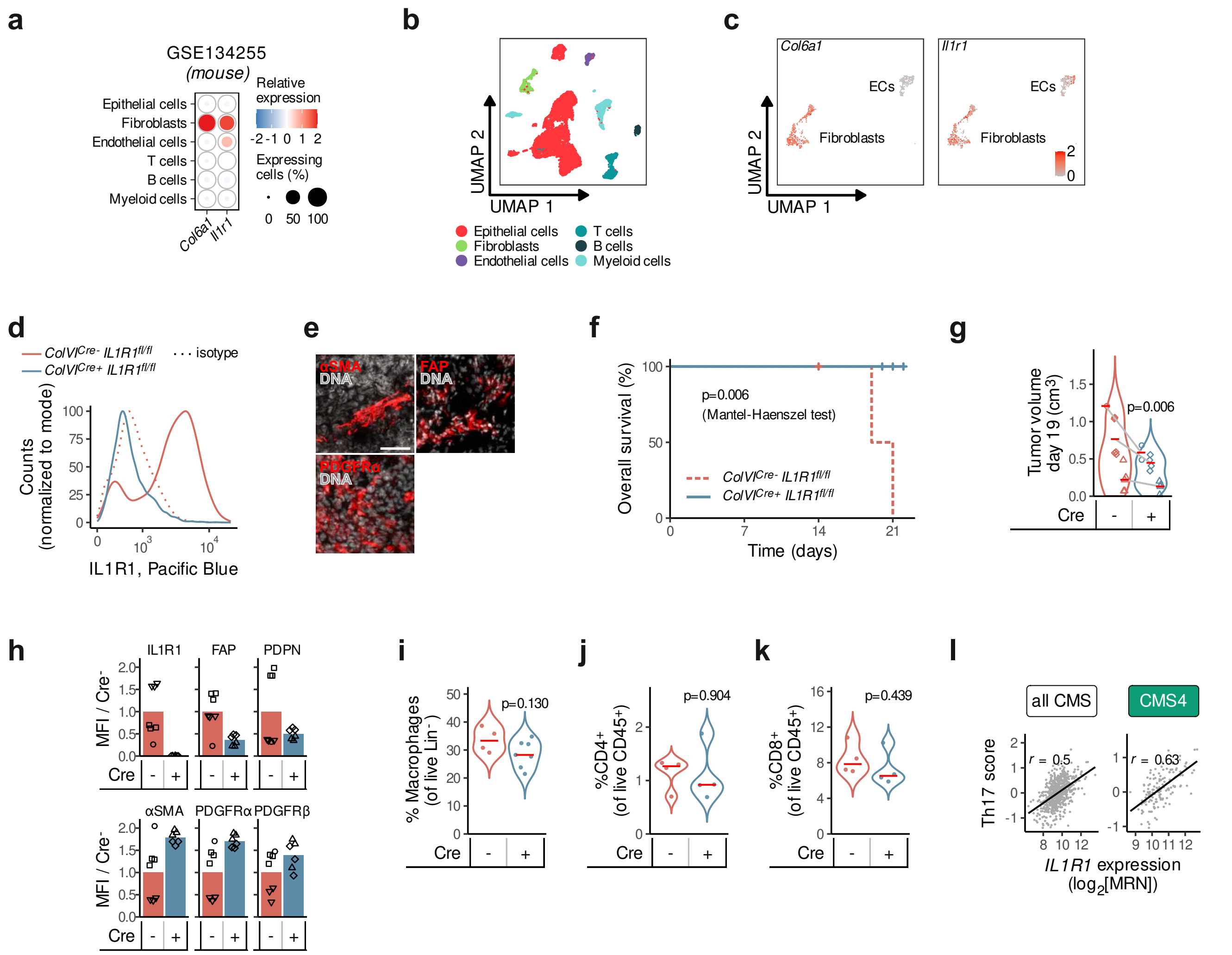

Supplementary Figure. 8 – Effect of the IL1R1⁺ iCAF subtype in an CRC mouse model. a Fibroblast specific expression of ColVI. Heatmap showing the expression of *Col6a1* and *Il1r1* in the main cell types of the mouse scRNA-seq dataset GSE134255 (n=7 mice). **b** UMAP plot showing the main cell types in GSE134255 (n=7 mice). **c** UMAP plot showing the expression of *Col6a1* and *Il1r1* in Fibroblasts and endothelial cells (ECs) in GSE134255 (n=7 mice). **d** IL1R1 expression in skin fibroblasts from ColVI^{cre+}IL1R1$^{fl/fl}$ and ColVI^{cre-}IL1R1$^{fl/fl}$ mice as measured by flow cytometry. n=3 mice. **e** Presence of fibroblasts in MC38 tumors shown by αSMA, FAP and PDGFRα immunofluorescence stainings as well as DAPI stained DNA content. Representative images out of n=3 tumors. Scale bar = 50 µm. **f** Kaplan-Meier curves showing the survival of ColVI^{cre+}IL1R1$^{fl/fl}$ (n=6 mice) and ColVI^{cre-}IL1R1$^{fl/fl}$ (n=7 mice) mice subcutaneously implanted with MC38 cells. P-value from a two-sided Mantel- Haenszel test is shown. **g** Tumor volumes (cm^3) at experimental endpoint from three independent experiments with n=11 mice per condition. Nested ANOVA. **h** Expression of CAF markers measured by flow-cytometry on colon fibroblasts isolated from *IL1R1* deficient (Cre$^+$, n=2 mice with number of technical replicates shown on the graph) and control (Cre$^-$, n=3 mice with number of technical replicates shown on the graph) group. **i-k.** Total of macrophages in **i**, CD4$^+$ cells in **j** and CD8$^+$ cells in **k** as assessed by flow cytometry. One representative experiment out of two are shown with n=4 Cre$^-$ and n=7 Cre$^+$ mice in **i** and n=4 mice per condition in **j-k**. **l** Correlation of *IL1R1* expression with Th17 scores in TCGA patients in all CMS (left panel, n=192) and CMS4 only (right panel, n=192). The Pearson's coefficient r is shown. Source data are provided as a Source Data file.

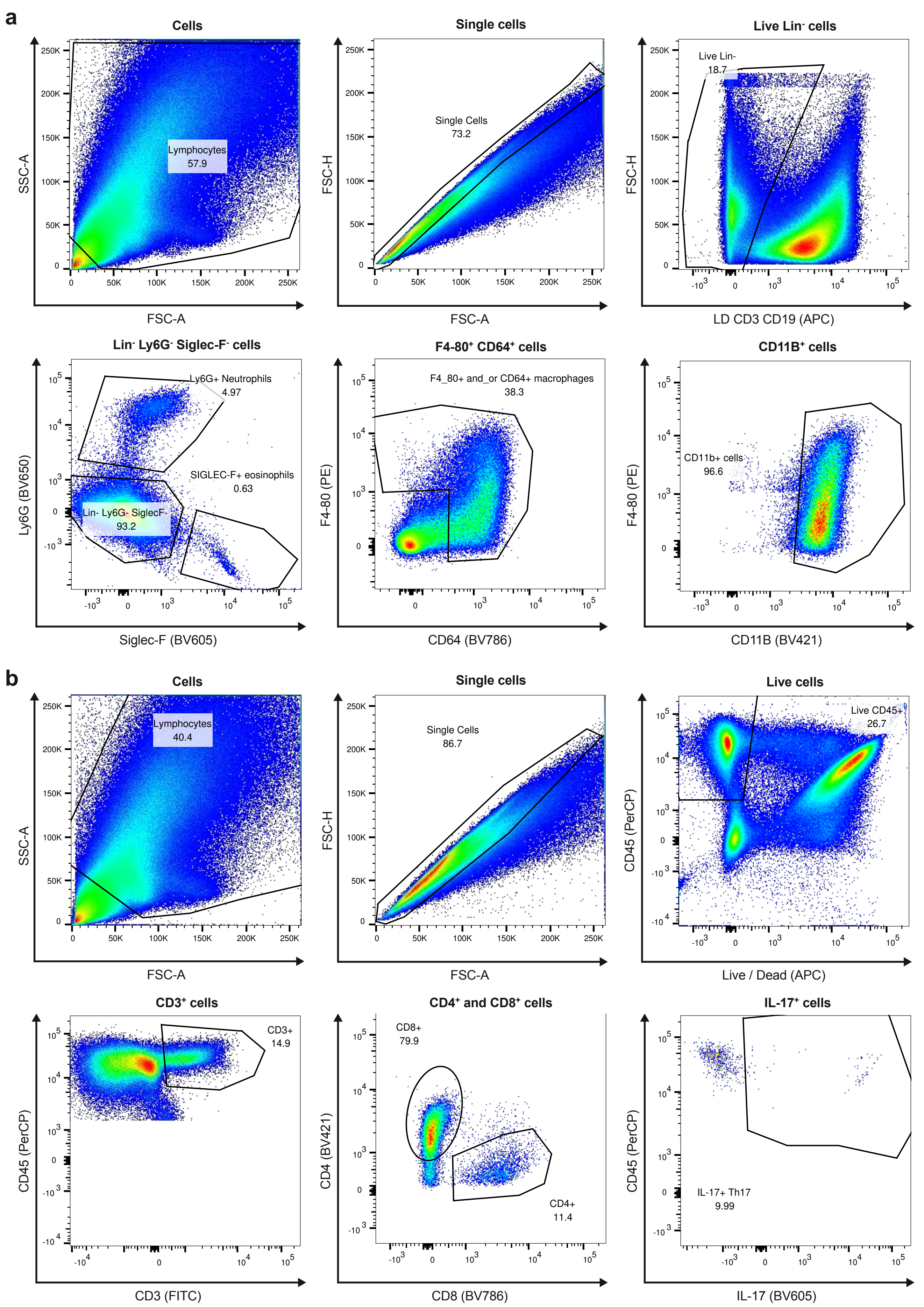

Supplementary Figure. 9 – Representative gating strategy of macrophages and lymphocytes. a Gating strategy for macrophages. **b** Gating strategy for CD4$^+$ and CD8$^+$ and IL-17$^+$ T-cells.

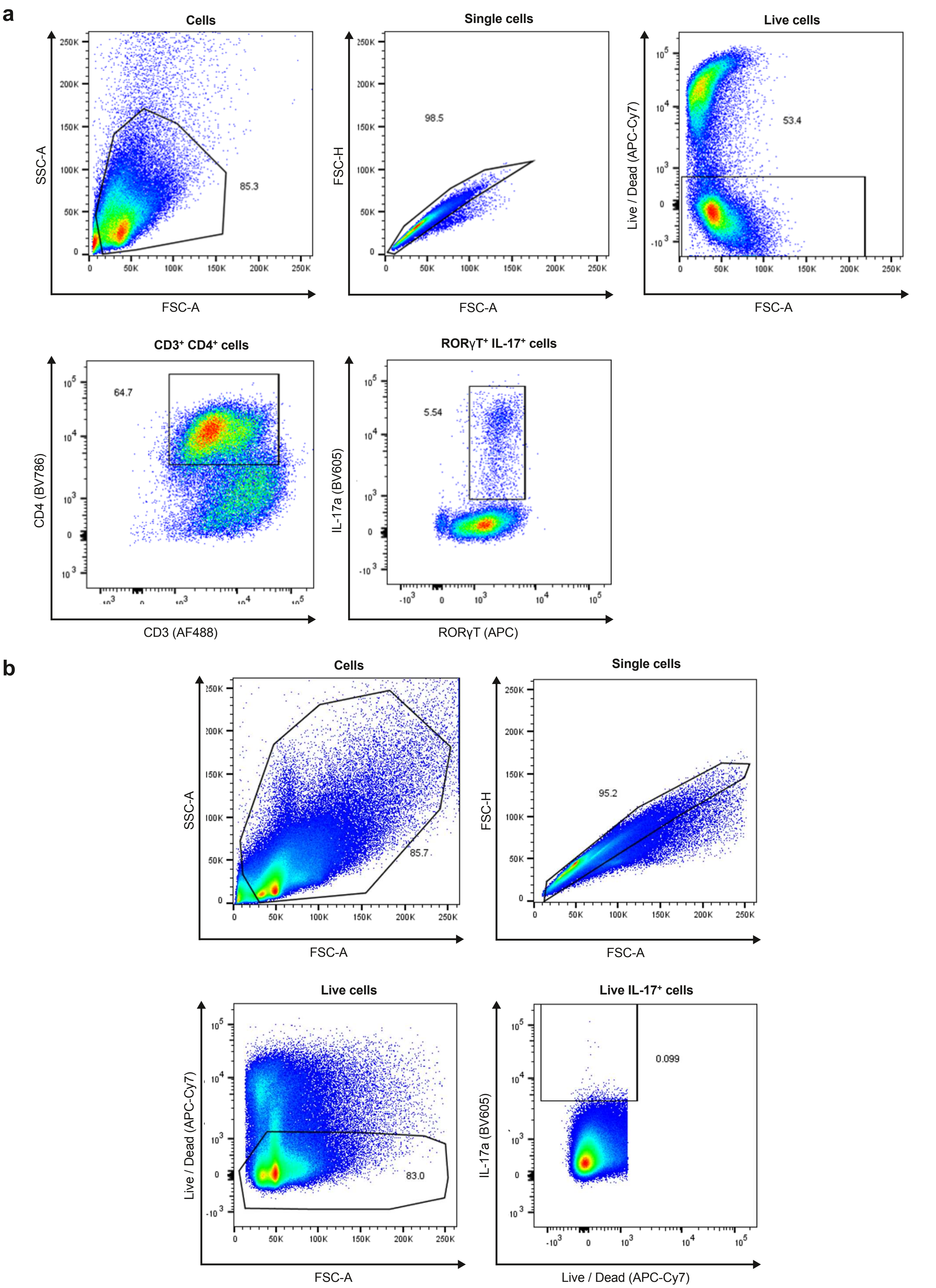

Supplementary Figure. 10 – Representative gating strategy of IL-17⁺ T cells. a Gating strategy for *in-vitro* T cell differentiation. **b** Gating strategy for *in-vivo* IL-17⁺ cells.

IL-1β

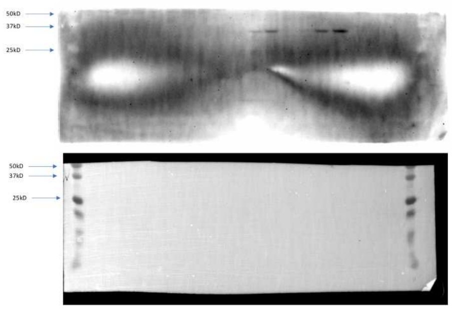

Actin

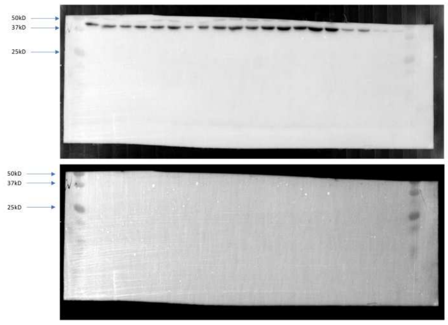

NFκB p65 (Santa Cruz; sc-109)
1/200 dilution

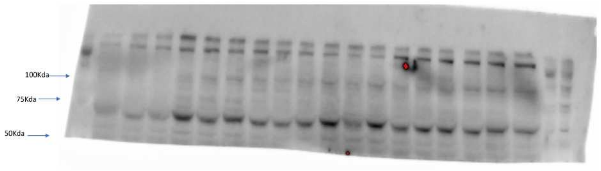

Phospho-NF-κB p65 (Cell Signalling; #3033P)
1/1000 dilution

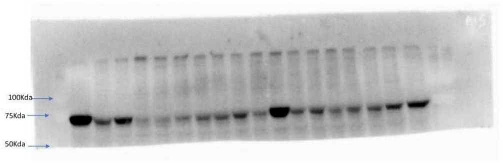

Actin (Millipore; MAB1501)
1/5000 dilution

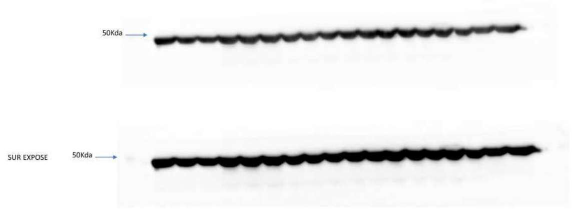

Supplementary Figure. 11 – Uncropped western blots. Raw western blot acquisitions shown in supplementary figure 4e.

Supplementary Table 1 – Patient characteristics of primary tumor derived fibroblast cell lines.

ID	Gender	TNM
P42	Female	pT3N0M0
P175	Male	n.a.
P177	Male	n.a.
P178	Female	n.a.
CAF-5	Female	pT2N0M0
CAF-6	Female	pT3N0M0
CAF-7	Male	pT2N0M0

Supplementary Table 2 – Gene symbols defining the IL-1β, iCAF and IL1R1$^+$ iCAF signatures.

signature	gene symbols
IL-1β	*CSF3, IL6, CCL20, CXCL2, CXCL8, CXCL3, CXCL10, CXCL1, SERPINB2, TNFAIP3, TNFAIP6, G0S2, EREG, CCL2, TNFAIP2, ICAM1, TNFSF15, IL1B, PTX3, IER3, ZC3H12A, INHBA, CXCL6, IL11, CXCL5, PTGS2, SLC7A2, SOD2, C11orf96, TSLP, SLC39A14, NFKB2, NAMPT, NFKBIZ, ZC3H12C, IL7R, RELB, SLC2A6, NFKBIA, NFKB1, VCAM1, RIPK2, FGF2, GFPT2, BMP2, LIF, SLC39A8, PTGES, NINJ1 and WTAP*
iCAF	*WNT5A, COL7A1, PDGFRA, CTHRC1 and CTSK*
IL1R1$^+$ iCAF	*FAP, PDPN, TPBG, SERPINE1, EFEMP1, PDGFRL and CLU*

Supplementary Table 3 – Descriptive statistics of our in-house CRC cohort (106 patients analysed in the TMA in Fig. 2).

variable	n
**Age**	
≤ 65	19
> 65	87
**Gender**	
female	32
male	74
**Stage**	
1	15
2	42
3	37
4	10
unknown	2
**Tumor localisation**	
proximal colon	36
distal colon	40
rectosigmoid	7
rectum	21
unknown	2
**CMS**	
CMS1	14 (15.9%)
CMS2	22 (25.0%)
CMS3	11 (12.5%)
CMS4	26 (29.5%)
NOLBL	15 (17.0%)
Not subtyped	18

Supplementary Table 4 – List of LR pairs identified by LIANA between IL1R1+ iCAFs and macrophages or T cells. The 20 top-ranked LR pairs with an aggregate score < 0.05 are shown for IL1R1+ iCAFs expressing the receptor (in) and the ligand (out).

	Macrophages		CD8+ T cells	
rank	in	out	in	out
1	C1QB→LRP1	SERPINE1→PLAUR	CCL5→ADRA2A	CXCL12→CXCR4
2	IL1B→IL1R1+IL1RAP	C3→ITGAX	CCL5→SDC4	MXRA5→CD69
3	SPP1→ITGA5+ITGB1	COL1A1→CD93	ITGB2→THY1	COL1A1→CD44
4	CD14→ITGB1	C3→ITGAM	GZMA→PARD3	COL1A2→CD44
5	SPP1→ITGAV+ITGB1	COL1A2→CD93		COL1A1→ITGA2+ITGB1
6	PTGS2→CAV1	COL1A1→CD44		COL3A1→ITGA2+ITGB1
7	APOE→LRP1	C3→C3AR1		COL1A1→ITGA11+ITGB1
8	SERPINA1→LRP1	COL1A2→CD44		COL1A1→ITGA3+ITGB1
9	PSAP→LRP1	BGN→LY96		COL6A2→CD44
10	SPP1→ITGAV+ITGB5	APOE→TREM2		COL3A1→ITGA11+ITGB1
11	ITGB2→THY1	C3→CD81		COL1A1→ITGA1+ITGB1
12	ICAM1→CAV1	HP→CD163		COL1A2→ITGA2+ITGB1
13	GNAI2→CAV1	DCN→TLR2		COL6A1→CD44
14	MMP9→LRP1	C3→NRP1		COL3A1→ITGA1+ITGB1
15	IL1B→IL1B+IL1R1+IL1RAP	COL1A1→CD36		COL1A2→ITGA11+ITGB1
16	GRN→TNFRSF1A	MXRA5→PILRA		COL1A2→ITGA1+ITGB1
17	NAMPT→ITGA5+ITGB1	FN1→C5AR1		COL1A2→ITGA3+ITGB1
18	C1QB→C1QBP	COL6A2→CD44		CXCL12→CXCR3
19	SPP1→ITGA4+ITGB1	SPON2→ITGB2		C3→IFITM1
20	IL6→IL6+IL6R+IL6ST	HP→ITGAM		MYL9→CD69
	CD4+ T cells		Tregs	
rank	in	out	in	out
1	LTB→TNFRSF1A	MXRA5→CD69	LTB→TNFRSF1A	COL1A1→CD44
2	CD40LG→ITGA5+ITGB1	CXCL12→CXCR4		COL1A2→CD44
3		COL1A1→CD44		CXCL12→CXCR4
4		COL1A2→CD44		MXRA5→CD69
5		COL1A1→ITGA11+ITGB1		COL3A1→ITGA11+ITGB1
6		COL6A2→CD44		COL1A1→ITGA11+ITGB1
7		COL3A1→ITGA11+ITGB1		COL1A1→ITGA3+ITGB1
8		COL6A1→CD44		COL1A2→ITGA11+ITGB1
9		COL1A2→ITGA11+ITGB1		COL1A2→ITGA3+ITGB1
10		VCAN→SELL		COL6A2→CD44
11		C3→IFITM1		COL6A1→CD44
12		APOE→SORL1		VCAN→SELL
13		COL3A1→ITGA1+ITGB1		C3→IFITM1
14		COL1A1→ITGA3+ITGB1		COL3A1→ITGA1+ITGB1
15		COL3A1→ITGA2+ITGB1		COL14A1→CD44
16		COL1A1→ITGA1+ITGB1		COL1A1→ITGA1+ITGB1
17		COL1A1→ITGA2+ITGB1		THBS2→ITGA4
18		COL14A1→CD44		B2M→CD3D
19		MYL9→CD69		ICAM1→IL2RA
20		COL1A2→ITGA3+ITGB1		ICAM1→IL2RG

Supplementary Table 5 – List of antibody references and primer sequences.

Antibodies	Manufacturer	Cat No	Dilution	Figure
**Mouse antibodies**				
IL1R1 /CD121a	BD Biosciences	564387	1:200	6C, S8H
Isotype control	BD Biosciences	562868	1:200	6C, S8D
CD3	BD Biosciences	553062	1:200	6F, S8I
CD4	BD Biosciences	563727	1:200	R
CD8	BD Biosciences	558106	1:200	6F, S8I
CD8	Invitrogen	67-0081-82	1:200	6F, S8I
CD45	BioLegend	103132	1:200	6F, S8I
IL17a	BD Biosciences	564169	1:100	6F, S8I, 6K
CD8	BioLegend	100712	1:200	5C
CD274 (PD-L1)	BD Biosciences	564715	1:200	6H
RORγT	eBioscience	17-6981-82	1:100	6K
CD3ε	BioLegend	100340	5 µg/ml	6G
CD28	BioLegend	102116	1 µg/ml	6G
IFN-γ	BD Pharmingen	554408	5 µg/ml	6G
αSMA	Cell Signaling	19245	1:200	S8E, S8H
PDGFRα	Abcam	ab61219	1:200	S8E, S8H
PDGFRβ	BioLegend	136006	1:200	S8H
FAP	Abcam	ab218164	1:200	S8E
FAP	R&D Systems	FAB9727R-100UG	1:200	S8H
PDPN	BioLegend	127422	1:200	S8H
aSMA	eBioscience	53-9760-82	1:200	S8H
PDGFRa	BD Biosciences	740148	1:200	S8H
CD11b	BioLegend	101224	1:200	S8I
F4/80	ThermoFisher Scientific	25-4801-82	1:200	S8I
CD64	BD Biosciences	741024	1:200	S8I
Ly6G	BD Biosciences	740554	1:200	S8I
SIGLEC-F	BD Biosciences	740388	1:200	S8I
CD19	ThermoFisher Scientific	A15391	1:200	S8I
CD3ε	ThermoFisher Scientific	47-0031-82	1:200	S8I
**Human antibodies**				
CD140a/PDGFRα	Abcam	ab124392	1:50	3H
CD140b/PDGFRβ	BD Biosciences	564124	1:200	3H
FAP	Abcam	ab53066	1:50 - 1:100	3H, S4I
IL1R1	R&D Systems	FAB269P	1:40	1D, S8D
IL1R1	Abcam	ab106278	1:50 - 1:500	3H
PDPN	BioLegend	337014	1:50	3H, 2J
CD274 (PD-L1)	BioLegend	329733	1:50	3H
αSMA	Invitrogen	53-9760-82	1:200	3H
FAP	Abcam	ab53066	1:100	2H
αSMA	Cell Signaling Technology	19245	1:500	2H
IL-1β	Abcam	ab2105	1:50 - 1:200	2H

PDPN	BioLegend	337022	1:50	2I, S4I
p65	Cell Signaling Technology	8242	1:1000	3G, S4F, S4H
CD140b/PDGFRβ	BioLegend	323606	1:100	S4I
αSMA	Abcam	ab184675	1:100	S4I
CD163	BioLegend	333608	1:50	5E
CD206	BioLegend	321116	1:100	5E
EPCAM	Cell Signaling Technology	5488	1:100	3G, S4F, S4H
VIM	Abcam	ab195878	1:5000	3G, S4F, S4H
**Viability dye**				
NearIR fluorescent reactive dye	Invitrogen	L34967	1:1000	3H,5C, 6F-G,6K,S8H
**Western blot antibodies**				
phospho-NF-κB p65	Cell Signaling Technology	3033P	1:1000	S4E
NF-κB p65	Santa Cruz	sc-109	1:200	S4E
IL-1β	Cell Signaling Technology	12242	1:1000	S4E
β-actin	Millipore	MAB1501	1:5000	S4E
rabbit-HRP	Cell Signaling Technology	7074	1:5000	S4E
mouse-HRP	Cell Signaling Technology	7076	1:5000	S4E

Primers	Sequences
IL8 - F	5'-ACTCCAAACCTTTCCACCCC-3'
IL8 - R	5'-ATTTCTGTGTTGGCGCAGTG-3'
IL1B - F	5'-CCACAGACCTTCCAGGAGAATG-3'
IL1B - R	5'-GTGCAGTTCAGTGATCGTACAGG-3'
IL6 - F	5'-AGACAGCCACTCACCTCTTCAG-3'
IL6 - R	5'-TTCTGCCAGTGCCTCTTTGCTG-3'
YWHAZ - F	5'-ACTTTTGGTACATTGTGGCTTCAA-3'
YWHAZ - R	5'-CCGCCAGGACAAACCAGTAT-3'
EFF1A1 - F	5'- TTGTCGTCATTGGACACGTAG-3'
EFF1A1 - R	5'- TGCCACCGCATTTATAGATCAG-3'
