# Supplementary material for: IL1R1+ cancer-associated fibroblasts drive tumor development and immunosuppression in colorectal cancer
Source: Nat Commun. 2023 Jul 17;14:4251. doi: 10.1038/s41467-023-39953-w (PMC10352362; doi:10.1038/s41467-023-39953-w)
Supplement: Supplementary file 3 — Description of Additional Supplementary Files [file 41467_2023_39953_MOESM3_ESM.pdf]

Description of Additional Supplementary Files

Title: Supplementary Data 1

Description: Metadata of the human datasets used in this study
