# Supplementary material for: IL1R1+ cancer-associated fibroblasts drive tumor development and immunosuppression in colorectal cancer
Source: Nat Commun. 2023 Jul 17;14:4251. doi: 10.1038/s41467-023-39953-w (PMC10352362; doi:10.1038/s41467-023-39953-w)
Supplement: Supplementary file 5 — Reporting Summary [file 41467_2023_39953_MOESM5_ESM.pdf]

nature portfolio

Corresponding author(s): Elisabeth Letellier

Last updated by author(s): Mar 1, 2023

Reporting Summary

Nature Portfolio wishes to improve the reproducibility of the work that we publish. This form provides structure for consistency and transparency in reporting. For further information on Nature Portfolio policies, see our Editorial Policies and the Editorial Policy Checklist.

Statistics

For all statistical analyses, confirm that the following items are present in the figure legend, table legend, main text, or Methods section.

n/a	Confirmed	
☐	☒	The exact sample size (*n*) for each experimental group/condition, given as a discrete number and unit of measurement
☐	☒	A statement on whether measurements were taken from distinct samples or whether the same sample was measured repeatedly
☐	☒	The statistical test(s) used AND whether they are one- or two-sided<br>*Only common tests should be described solely by name; describe more complex techniques in the Methods section.*
☒	☐	A description of all covariates tested
☐	☒	A description of any assumptions or corrections, such as tests of normality and adjustment for multiple comparisons
☐	☒	A full description of the statistical parameters including central tendency (e.g. means) or other basic estimates (e.g. regression coefficient) AND variation (e.g. standard deviation) or associated estimates of uncertainty (e.g. confidence intervals)
☐	☒	For null hypothesis testing, the test statistic (e.g. *F*, *t*, *r*) with confidence intervals, effect sizes, degrees of freedom and *P* value noted<br>*Give P values as exact values whenever suitable.*
☒	☐	For Bayesian analysis, information on the choice of priors and Markov chain Monte Carlo settings
☐	☒	For hierarchical and complex designs, identification of the appropriate level for tests and full reporting of outcomes
☐	☒	Estimates of effect sizes (e.g. Cohen's *d*, Pearson's *r*), indicating how they were calculated

Our web collection on statistics for biologists contains articles on many of the points above.

Software and code

Policy information about availability of computer code

Data collection

WB: detection was performed with the Fusion FX imaging platform
Flow Cytometric analysis and cell sorting were performed using BD FACSCantoll, BD FACSFortessa and BD FACSMelody
Incucyte Live-Cell Analysis System (Essen Bioscience)
Real-Time PCR system (Applied Biosciences)
RNA sequencing: RNA library quantity and quality were assessed with the Agilent 2100 BioAnalyzer (Agilent, USA). RNA libraries were sequenced as l00bp paired-end runs on an Illumina HiSeq2500 platform.
single cell sequencing, Dropseq

Data analysis

Image studio lite software for image anaylsis (WB)
Image J 1.53K
FlowJo software 10.6.1
BD FACSDiva (8.0.1)
BD FACSChorus
Cedex
R studio (2023.03.1 Build 446) with R CRAN (4.2.1)
R packages: tidyverse (2.0.0), targets (0.14.3), tarchetypes (0.7.5), ggbeeswarm (0.7.1), Seurat (4.3.0), clustree (0.5.0), SingleR (2.0.0), LIANA (0.1.5), DESeq2 (1.38.3), clusterProfiler (4.6.2), lme4 (1.1-32), lmerTest (3.1-3), rstatix (0.7.2) and ggpubr (0.6.0).
Incucyte ZOOM software (2018B, Sartorius, Germany)
Cytation Gen5

RT-qPCR data was analyzed using qBase+ 3.2 (Biogazelle) according to MIQE guidelines

For manuscripts utilizing custom algorithms or software that are central to the research but not yet described in published literature, software must be made available to editors and reviewers. We strongly encourage code deposition in a community repository (e.g. GitHub). See the Nature Portfolio guidelines for submitting code & software for further information.

Data

Policy information about availability of data

All manuscripts must include a data availability statement. This statement should provide the following information, where applicable:

- Accession codes, unique identifiers, or web links for publicly available datasets
- A description of any restrictions on data availability
- For clinical datasets or third party data, please ensure that the statement adheres to our policy

All Sequencing data has been uploaded to EGA and is available under the accession number EGAS00001007205.

The scRNA-Seq and RNA-Seq data generated in this study have been deposited at the European Genome-phenome Archive (EGA) under the accession number EGAS00001007205 [https://ega-archive.org/studies/EGAS00001007205]. The sequencing data generated in this study is of human origin. Hence, according to the European Data Protection Regulation, the data are available under restricted access, access can be obtained by contacting the corresponding author at Elisabeth.letellier@uni.lu. The procedure to access data is described in https://ega-archive.org/access/data-access.

The contact person is Prof. Elisabeth Letellier
Email: elisabeth.letellier@uni.lu
An interested scientist can contact Prof E. Letellier and after filling a form from the University of Luxembourg and once the application is approved, EGA will create an unique EGA-account that grants access to this one dataset. The whole procedure takes around 2 weeks.

The TCGA analysis was based upon data (TCGA-COAD, TCGA-READ) generated the TCGA Research Network: https://www.cancer.gov/tcga.

All scripts and codes are available under gitlab of the University of Luxembourg https://gitlab.lcsb.uni.lu/mdm/koncina_et_al_2023

Of note, one can login to the Gitlab instance with a standard Github account.

Human research participants

Policy information about studies involving human research participants and Sex and Gender in Research.

Reporting on sex and gender	Whenever available or relevant, information on sex and gender are indicated in the Figure legends or Material and Methods section. Gender data from the cohort is included in the Supplementary Table 2.
Population characteristics	All characteristics of the patients in the cohort are listed in Supplementary Table 2.
Recruitment	Patients are recruited as part of an open colorectal cancer sample collection associated to the secondary use for specific research projects.
Ethics oversight	All patient samples were obtained through our established colorectal cancer cohort and handled according to all institutional guidelines and regulations (ERP-16-032), based on previously described biospecimen handling standards.<br>All patient samples were donated freely, with informed consent and with the full cooperation of each patient. Ethical approval were obtained for samples collected in Luxembourg and Vienna from the Comité National d'Ethique de Recherche (CNER) (Reference 201009/09) and the ethics committee of the Medical University of Vienna (EK Nr: 1580/2017), respectively. We are not aware of any selection bias that may have impacted the results.

Note that full information on the approval of the study protocol must also be provided in the manuscript.

Field-specific reporting

Please select the one below that is the best fit for your research. If you are not sure, read the appropriate sections before making your selection.

☒ Life sciences ☐ Behavioural & social sciences ☐ Ecological, evolutionary & environmental sciences

For a reference copy of the document with all sections, see nature.com/documents/nr-reporting-summary-flat.pdf

Life sciences study design

All studies must disclose on these points even when the disclosure is negative.

Sample size	For in vitro studies a minimum of triplicates per condition were used and each experiment was verified in at least three biological replicates.

	As CAFs are short-term cultures, we worked with different CAF cultures to generate independent experiments. For in vivo work, the number of mice per group was calculated based on a power calculation in liaison with a bio-statistician. For subcutaneous tumor injections and based on two-sided t-test power calculations (power = 0.8, alpha = 0.05, sd = 1), n = 8 and n = 11 mice are required for mean differences of 0.3 and 0.4 respectively. Please refer to the Figure legends and Method sections for further details.
Data exclusions	no data was excluded unless clear technical issues were identified
Replication	All in vitro experiments were verified in at least three independent experiments or coming from three independent patient cultures with same overall outcome. Specific number of repetitions is provided in the manuscript. For in vivo experiments, adequate group size was calculated beforehand. As stated in the manuscript, for some experiments, we pooled two or three independent experiments to reach the number calculated by the bioinformatician. All in vivo replication assays were successful.
Randomization	Before starting all of the in vivo experiments, mice were randomly allocated to different groups.
Blinding	Analyses of tumor measurement were performed blindy. IDs were uncovered after final result was obtained.

Reporting for specific materials, systems and methods

We require information from authors about some types of materials, experimental systems and methods used in many studies. Here, indicate whether each material, system or method listed is relevant to your study. If you are not sure if a list item applies to your research, read the appropriate section before selecting a response.

Materials & experimental systems

n/a	Involved in the study
☐	☒ Antibodies
☐	☒ Eukaryotic cell lines
☒	☐ Palaeontology and archaeology
☐	☒ Animals and other organisms
☒	☐ Clinical data
☒	☐ Dual use research of concern

Methods

n/a	Involved in the study
☒	☐ ChIP-seq
☐	☒ Flow cytometry
☒	☐ MRI-based neuroimaging

Antibodies

Antibodies used	The antibodies used are listed in the Supplementary Table 3.
Validation	All antibodies used are well described, validated with positive controls and used across many studies and laboratories.

Eukaryotic cell lines

Policy information about cell lines and Sex and Gender in Research

Cell line source(s)	Most of the cell epithelial cells, normal and cancer-associated fibroblast matching culture lines used in this study were derived directly from patients tissue. CAF05 were purchased from Neuromics and maintained in endothelial growth media. Immortalized primary human colon fibroblasts (CT5.3) were provided by Prof. Olivier De Wever. MC38 (mouse tumor cells) were obtained from Kerafast and cultured according to the supplier protocol. CT26 (mouse tumor cells) and L-cells (normal mouse fibroblasts) were obtained from ATCC and are cultured as indicated by the provider.
Authentication	All regular ATCC cultures have been authenticated by STR profiling.
Mycoplasma contamination	All samples have been checked for Mycoplasma contamination before the start of the experiments.
Commonly misidentified lines (See ICLAC register)	No commonly misidentified lines were used

Animals and other research organisms

Policy information about studies involving animals; ARRIVE guidelines recommended for reporting animal research, and Sex and Gender in Research

Laboratory animals	In this study we used Mus musculus ColVIcre+IL1R1fl/fl and ColVIcre-IL1R1fl/fl on the C57Bl/6J background. Additionally wild-type BALB/cwere used. ColVIcre+ mice were a kind gift from Dr. George Kollias35 and IL1R1fl/fl mice were obtained from The Jackson Laboratory.<br>Animals were aged 8-12 weeks and maintained under standard habitat conditions (humidity: 40–70%, temperature: 22 °C) with a 12:12 light cycle.
Wild animals	No use of wild animals.

Reporting on sex	We used both genders as stated in the Material and Methods part of the manuscript.
Field-collected samples	No field collected samples were used
Ethics oversight	Animal experiments were performed according to all applicable laws and regulations, after approval by the Animal Experimentation Ethics Committee (AEEC) of the University of Luxembourg, as well as by the Luxembourgish Ministry of Agriculture, Viniculture & Rural Development (LUPA 2020/10), in accordance with the EU Directive 2010/63/EU, as well as the Grand-Ducal Regulation of 11 January 2013 (2013-1-11) on the protection of animals used for scientific purposes. These included the justification of the use of animals, their welfare and the incorporation of the principles of the "3Rs" (Replacement, Reduction and Refinement). All experiments were carried out with veterinarian consent and all animal protocols were reviewed by a biostatistician. Humane endpoint (HEP) is defined by the AEEC as the combined size of tumors on both flanks not surpassing 2000 mm3. This limit was not exceeded in our experiments.

Note that full information on the approval of the study protocol must also be provided in the manuscript.

Flow Cytometry

Plots

Confirm that:

- [x] The axis labels state the marker and fluorochrome used (e.g. CD4-FITC).
- [x] The axis scales are clearly visible. Include numbers along axes only for bottom left plot of group (a 'group' is an analysis of identical markers).
- [ ] All plots are contour plots with outliers or pseudocolor plots.
- [x] A numerical value for number of cells or percentage (with statistics) is provided.

Methodology

Sample preparation	Single cell suspension was stained with live/dead dye and with the corresponding antibody panels (Supplementary Table 4) for 30 min at 4°C. For the intracellular staining the Cytofix/Cytoperm™ Fixation/Permeabilization Kit (554714, BD Biosciences) was used.<br>Acquisition was performed on a BD FACSCantoll and a BD FACSFortessa machine and data was analyzed using FlowJo software 10.6.1. FACS sorting was performed using BD FACS Melody.
Instrument	BD FACS Cantoll, BD LSR Fortessa FACS machine and BD FACS Melody cell sorter
Software	BD FACS Diva 8.0.1, BD FACS Chorus 3.0 and FlowJo 10.6.1 softwares
Cell population abundance	Total cell counts were determined by Cedex and multiplied by frequency of subpopulations to get absolute counts.
Gating strategy	Populations of interest were gated based on size and granularity in the initial FSC-A vs SSC-A gate. Doublet exclusion was performed by eliminating non-linear ratios of FCS-A and FSC-H in the subsequent gate. Dead cells were then excluded based on viability dye stain. Further gates were set according to clearly visible populations(as shown in the supplementary data).<br>For the phenotypical characterization of fibroblasts, where markers are differentiated by level of expression instead of clear populations, mean fluorescence intensity as used.

- [x] Tick this box to confirm that a figure exemplifying the gating strategy is provided in the Supplementary Information.
